# Lennox-Gastaut syndrome characterized by super-refractory status epilepticus treated with high-dose anesthetics: A case report

**DOI:** 10.1097/MD.0000000000035233

**Published:** 2023-09-29

**Authors:** Xiaoqian Yang, Guangjun Xu, Zonglei Chong, Yangyang Liang, Jingwei Du, Lin Zhao, Wei Chen

**Affiliations:** a Department of Neurology, Liaocheng People’s Hospital, Liaocheng, Shandong Province, P.R. China; b Department of Neurosurgery, Liaocheng People's Hospital, Liaocheng, Shandong Province, P.R. China; c Department of Neurology, Dong'e County People’s Hospital, Donge County, Liaocheng, Shandong Province, P.R. China.

**Keywords:** burst, dose anesthetic, Gastaut syndrome, high, Lennox, refractory status epilepticus, super, suppression

## Abstract

**Rationale:**

Super-refractory status epilepticus is a serious illness with high morbidity and mortality, which is defined as an SE that continues or recurs 24 hours or more after the onset of anesthesia. Anesthetic agents can be either pro-convulsant or anticonvulsant or both.

**Patient concerns:**

Epilepsy occurred at the age of 3 years. At the age of 4 years, generalized tonic-clonic seizure occurred for the first time. The patient was hospitalized at the age of 27 and 28 years for treating status epilepticus. At the age of 33 years, antiepileptic drugs were stopped due to poor appetite. In an early morning, the patient was found delirious with reduced speech.

**Diagnosis:**

Occasionally, the patient blinked his eyelids, or deflected his eyeballs to 1 side. When propofol was lowered to 10 mL/H, the epileptic wave reduced obviously. Afterwards, the patient opened his eyes autonomously and his consciousness gradually recovered. The patient could answer questions, and the limbs had voluntary movements. The patient breathing also gradually recovered, and his urine gradually returned to pale yellow from green. After anesthetic was stopped for 10 days, the patient lost his consciousness again. The patient eyes turned upward frequently, which was relieved in 1 to 2 seconds with an attack once every 2 to 5 minutes.

**Interventions:**

Clonazepam was gradually reduced to 2 mg qn, and the patient gradually woke up during this process. The patient was also treated with levetiracetam 1.5 g bid, oxcarbazepine 0.6 g bid, topiramate 50 mg bid and valproate 0.4 g tid.

**Outcomes:**

After 1 month follow-up, status epilepticus did not appear again.

**Lessons:**

Propofol aggravated the tonic seizures. As tonic seizures occur during natural sleep and after sleep induced by various narcotic drugs, the decrease of consciousness level induced by excessive sedation of narcotic drugs has been suggested as the reason for poor seizure control.

## 1. Introduction

A 33-year-old male with Lennox-Gastaut syndrome (LGS) characterized by super-refractory status epilepticus (SRSE) got worse after the application of high-dose anesthetic. Burst-suppression in SRSE cannot be used as a guideline for withdrawal of high-dose anesthetics. To the best of our knowledge, this study for the first time proposed that high dose propofol could aggravate tonic seizures in LGS. The full-conduction low-amplitude fast-wave activity of electroencephalogram (EEG) is possibly a sign before the occurrence of tonic status epilepticus (SE). Benzodiazepines should be considered as the first-line drugs for the treatment of tonic seizures.

SRSE is a serious illness with high morbidity and mortality,^[1–3]^ which is defined as a SE that continues or recurs 24 hours or more after the onset of anesthesia. SRSE also includes the cases in which SE recurs on the reduction or withdrawal of anesthetics.^[4]^ The main goal of treatment is to stop seizure activity and improve clinical outcomes.^[5,6]^ Anesthetic agents and anticonvulsants are the most used drugs in the treatment of SE. Some alternatives that have been commonly applied in the refractory cases include lesion resection, implantation of neuromodulator, ketogenic diet, pyridoxine infusion, cerebrospinal fluid drainage and magnesium infusion.^[4]^ Anesthetic agents can either be pro-convulsant or anticonvulsant, or both.^[7]^ Here, we reported the case of a 33-year-old male with LGS, who was characterized by the deterioration of SRSE after the application of high-dose anesthetics.

## 2. Case report

**Figure 1.**
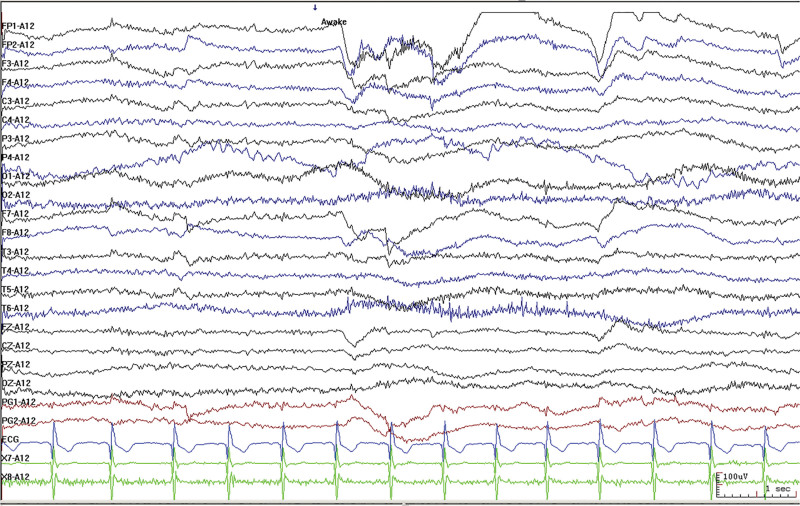
Extensive fast wave rhythm of electroencephalogram (EEG) in all channels.

**Figure 2.**
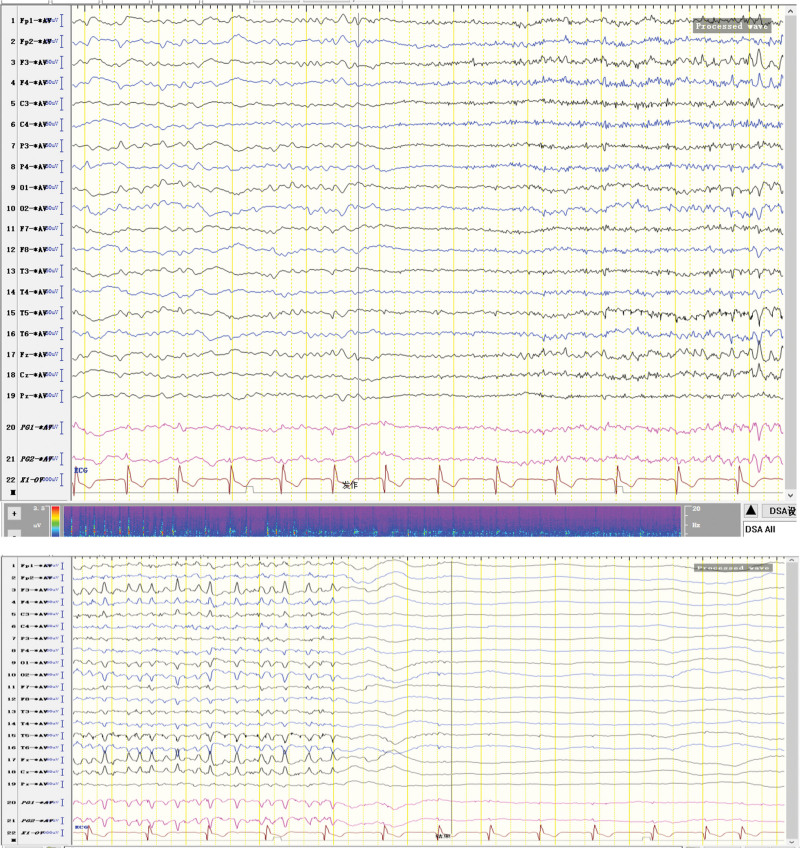
Voltage dropped in all channels of electroencephalogram (EEG) → Extensive low-amplitude fast wave rhythm → The amplitude gradually rose and the frequency gradually slowed down → Extensive medium-high amplitude fast wave and spike wave rhythm.

**Figure 3.**
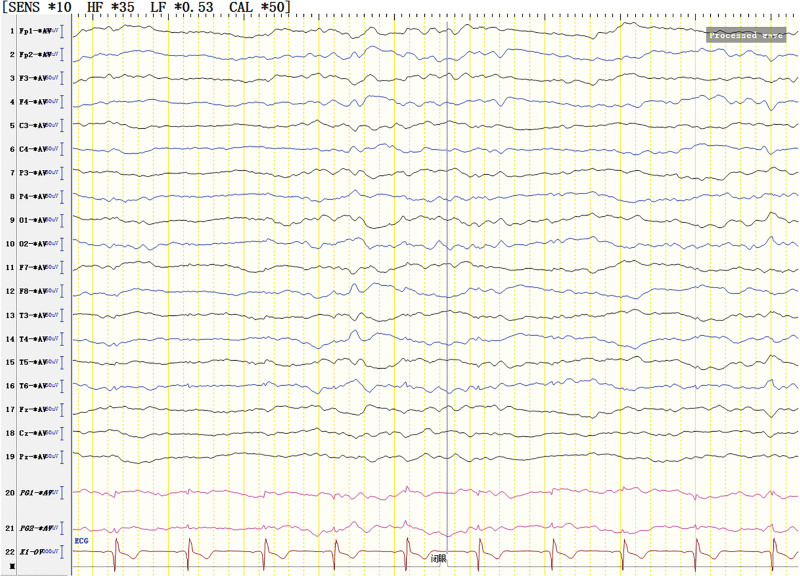
Burst-suppression trend. Burst section: Diffuse irregular slow waves with medium-high amplitude in all channels of electroencephalogram (EEG) (The θ waves dominated). Sharp waves were mixed among them. Suppression section: voltage <20 uv, lasting for 1 s to 7 s.

**Figure 4.**
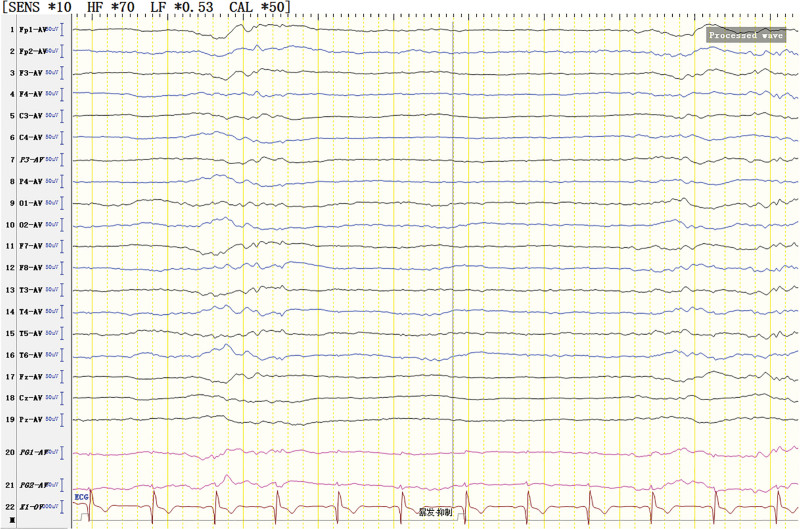
Burst-suppression trend. Burst section: Diffuse irregular slow waves with low-medium amplitude in all channels of electroencephalogram (EEG) (The θ waves dominated). Sharp waves were mixed among them. Suppression section: voltage <5 uv.

**Figure 5.**
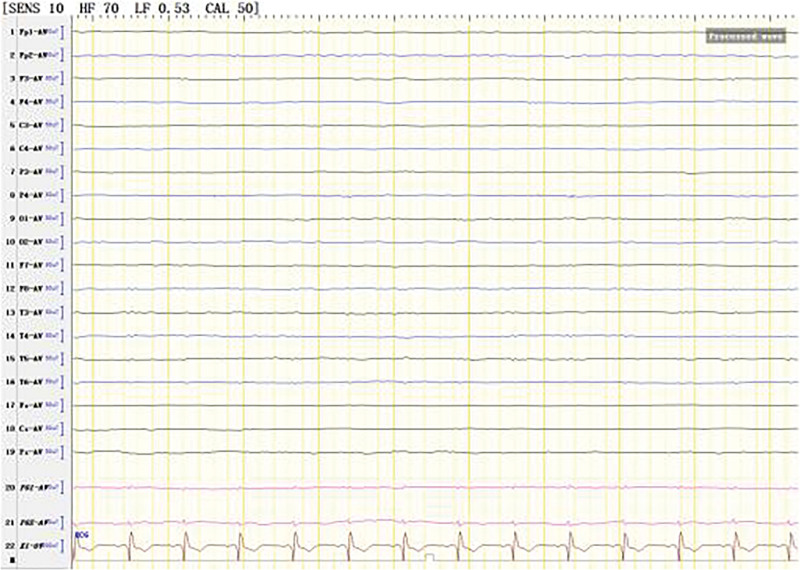
Generalized electroencephalogram (EEG) suppression (voltage <2 uv).

**Figure 6:**
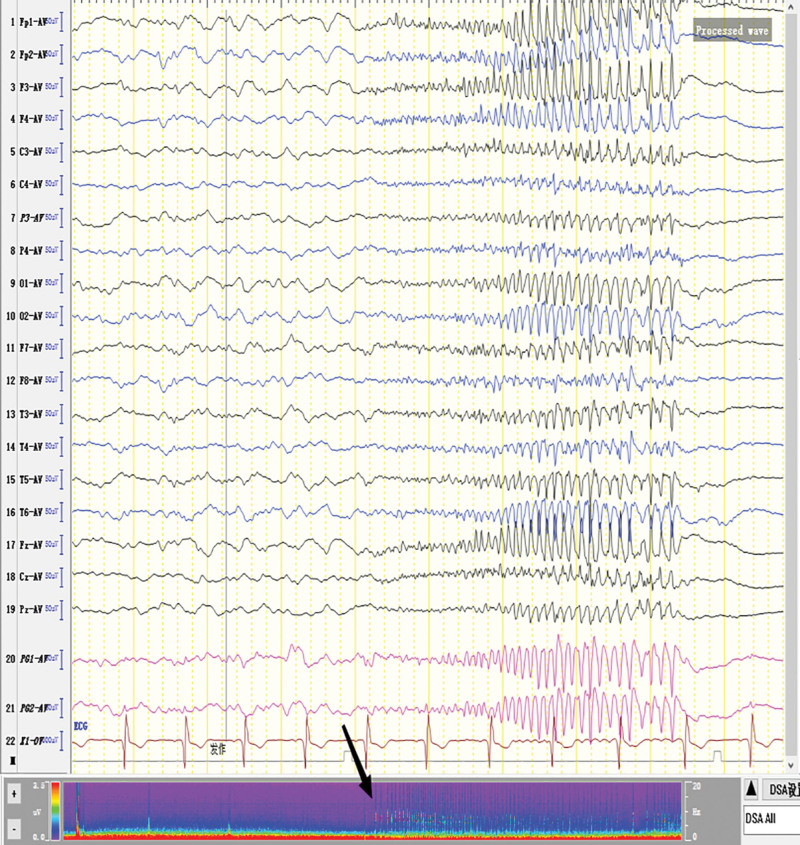
. The number of seizures began to apparently increase (as the arrow points out).

**Figure 7.**
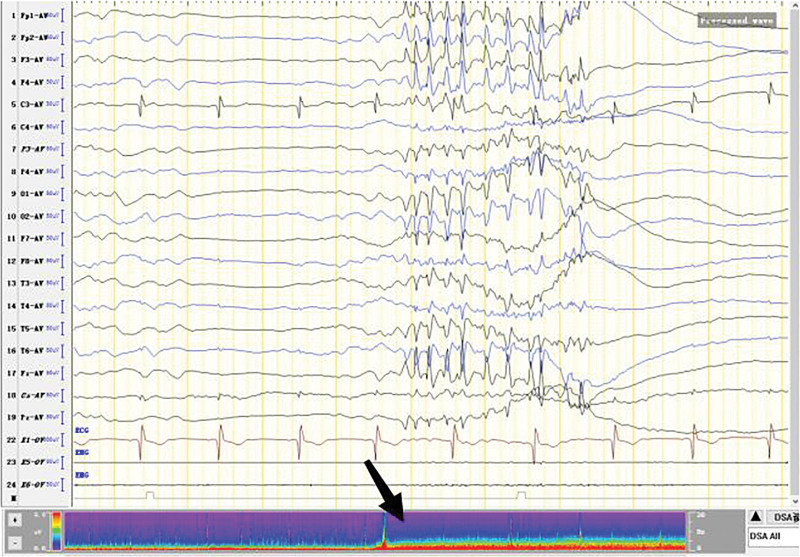
The number of seizures decreased after reducing the intravenous injection speed of propofol (as the arrow points out).

**Figure 8.**
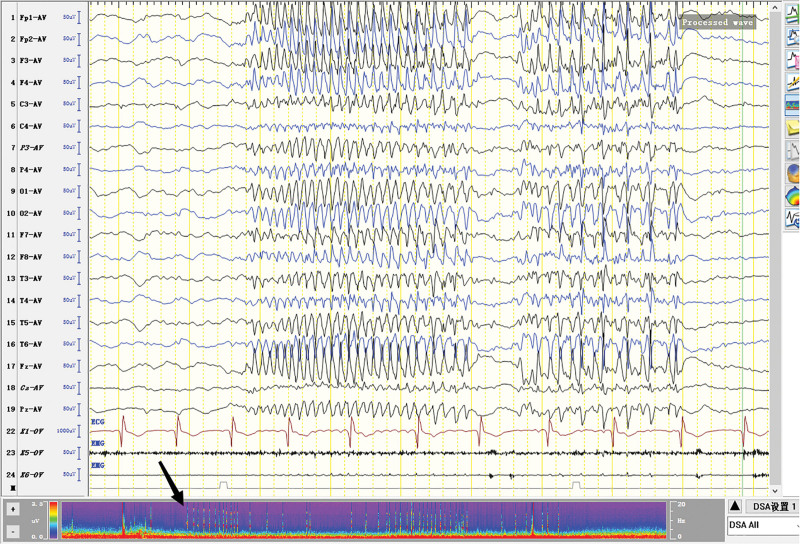
The frequency of seizures increased with the increase of intravenous injection speed of propofol (as the arrow points out).

**Figure 9.**
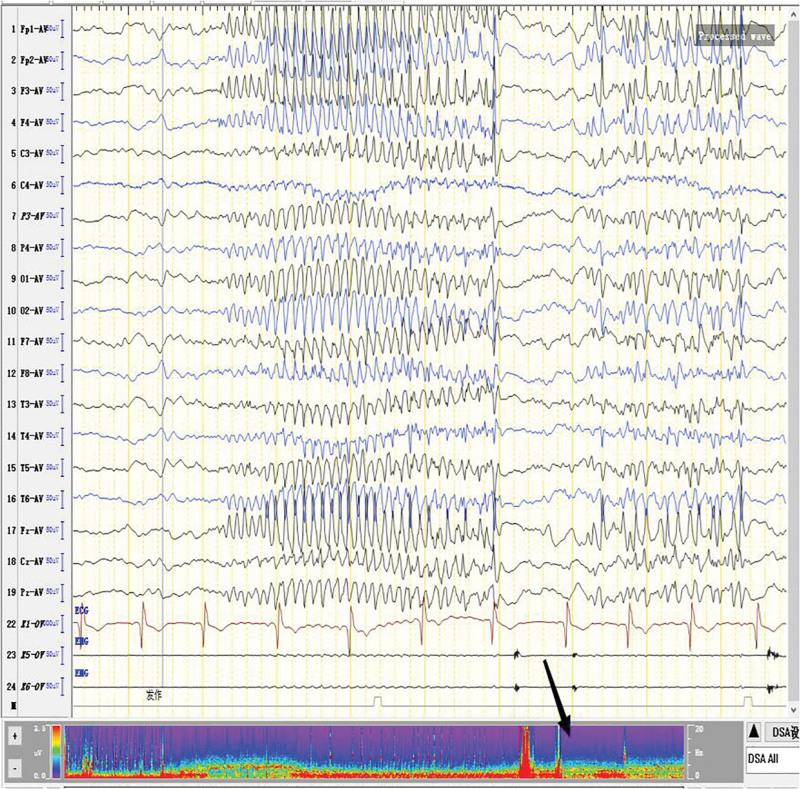
After reducing the intravenous injection speed of propofol again, the number of seizures was apparently reduced (as the arrow points out).

The patient was born in 1989. Epilepsy occurred at the age of 3 years, characterized by a sudden fall followed by convulsions. At the age of 4 years, generalized tonic-clonic seizure (GTCS) occurred for the first time. The patient was hospitalized at the age of 27 and 28 years for treating SE. The EEG signals showed a lot of sharp slow waves, sharp waves and slow waves in the bilateral cerebral hemispheres. The patient was treated with oral oxcarbazepine and sodium valproate. He was inferior to his peers in intelligence since childhood. At the age of 33 years, antiepileptic drugs were stopped due to poor appetite. Afterwards, poor epilepsy control occurred. In an early morning, the patient was found delirious with reduced speech. The EEG signals showed a wide range of fast wave rhythms (Fig. 1). The patient did not respond to pain stimulation, but pathological signs were negative. After 34 hours, the patient developed coma, accompanied by paroxysmal elevation of both upper limbs, flexion of both hips and knees, lifting of the head and turning up of the eyes. The patient signed the informed consent for treatment. All procedures carried out on the patient complied with the Helsinki Declaration. Written informed consent of the patient was obtained for this case report. After intravenous injection of diazepam, midazolam and valproate, and intramuscular injection of phenobarbital, the frequency of epilepsy decreased to once every 15 to 20 minutes, lasting for 3 to 5 seconds. He was then transferred to the intensive care unit and received auxiliary ventilation. At this time, the patient was diagnosed with selenium poisoning, and was given levetiracetam, valproate and oxcarbazepine by gastric tube injection, as well as midazolam and propofol by intravenous injection. The EEG signals showed frequent tonic episodes (Fig. 2). The patient frequently stared upward in both eyes, lasting 10 seconds each time, with an attack every 7 to 15 minutes. We added levetiracetam with a dose of 1 g bid, valproate with a dose of 1.2 g bid. The injection rate of midazolam was adjusted to 20 mg/H and propofol was adjusted to 0.15 g/H. The EEG signals showed burst-suppression (Fig. 3). Afterwards, the patient had frequent tonic attacks, respiratory depression and hypotension. We gradually reduced the dosage of midazolam and adjusted the dosage of valproate to 0.4 g tid. We also gradually increased the pump speed of propofol to 0.2 g/H. The EEG signals showed lower amplitude and shorter duration of bursts with an inhibitory segment (i.e., a prolonged duration of 17 seconds and a voltage of <5 uv) (Fig. 4). There was no improvement in the frequency of ocular gaze onset. After intravenous injection of 100 mg propofol, the EEG signals were completely suppressed for 6 minutes (Fig. 5), but then went back to burst-suppression. After propofol infusion speed was increased to 0.6 g/H, the epileptic wave elevated apparently (Fig. 6). Since the patient had stopped breathing spontaneously, the intravenous rate of propofol was reduced to 0.4 g/H, and the epileptic waves apparently reduced (Fig. 7). The EEG signals showed that interictal epileptiform discharges recovered. Physiological waveforms showed a continuous pattern, and the burst-suppression decreased. When the intravenous infusion rate of propofol was increased to 0.6 g/H again, the epileptic wave increased (Fig. 8). Occasionally, the patient blinked his eyelids, or deflected his eyeballs to 1 side. When propofol was lowered to 10 mL/H, the epileptic wave reduced obviously (Fig. 9). Afterwards, the patient opened his eyes autonomously and his consciousness gradually recovered. The patient could answer questions, and the limbs had voluntary movements. The patient breathing also gradually recovered, and his urine gradually returned to pale yellow from green.

After anesthetic was stopped for 10 days, the patient lost his consciousness again. The patient eyes turned upward frequently, which was relieved in 1 to 2 seconds with an attack once every 2 to 5 minutes. The patient was diagnosed with SRSE. Afterwards, propofol was given intravenously (0.6 g/H), and clonazepam was given through gastric tube (2 mg qn). Other oral drugs were not adjusted. The eyes turned upward less frequently. The EEG signals showed burst-suppression. The voltage range of EEG burst was <20 uv. The duration of inhibitory segment of EEG was prolonged to 13 seconds. A few hours later, the patient condition worsened. The eyes turned upward more frequently, and the duration became longer, which was subsequently relieved by an increase of clonazepam to 2 mg bid. After twenty-four hours, no seizure or epileptic wave occurred, and propofol was reduced at a rate of 5 mL/3H. The EEG signals showed that epileptic waves increased significantly when propofol was reduced to 0.2 g/H. The propofol was not further reduced, while oxcarbazepine was added to 0.3 g bid and clonazepam was added to 2 mg tid.

**Figure 10.**
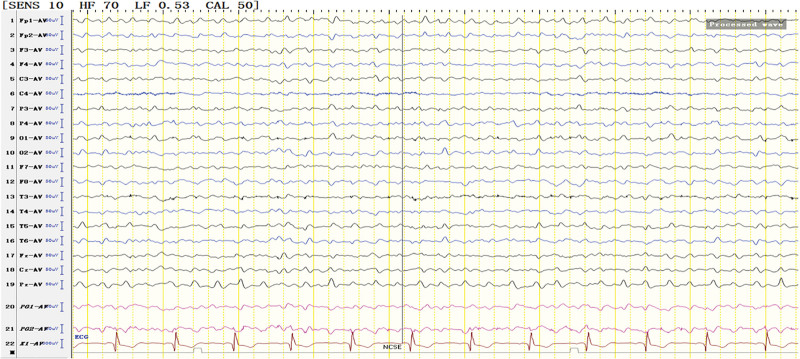
Medium-high amplitude sharp waves and sharp slow waves were continuously emitted in the midline frontal region, bilateral frontal poles, frontal and anterior temporal regions.

The patient condition was not improved and had 2 GTCSs, each lasting about 10 minutes. Afterwards, tonic status epilepticus was alternated with non-convulsive status epilepticus (NCSE) (Fig. 10). The patient was treated with topiramate 25 mg bid. Clonazepam was gradually added to 4 mg q8h. Oxcarbazepine was added to 0.6 g bid, and propofol was injected intravenously at a rate of 0.2 g/H. After 3 days, propofol was reduced to 5 mL/3H. The patient still frequently turned his eyes upward. Due to unconsciousness of the patient, clonazepam was gradually reduced to 2 mg qn, and the patient gradually woke up during this process. The patient was also treated with levetiracetam 1.5 g bid, oxcarbazepine 0.6 g bid, topiramate 50 mg bid and valproate 0.4 g tid. Finally, condition of the patient improved apparently. The eyes were turned upward once every 1 to 2 days. When falling asleep, the EEG signals showed tonic seizures, and the patterns of GTCS and NCSE did not appear again. The patient was hospitalized for 56 days and then discharged. After 1 month follow-up, SE did not appear again.

## 3. Discussion

For the treatment of SRSE, the therapeutic goal of anesthetic is to stop SE. It remains unclear about the depth of anesthesia and the timing of reducing anesthetics.^[8]^ It has been suggested that the dosage of anesthetic should be gradually increased until EEG burst inhibition occurs.^[9–11]^ Afterwards, the dosage of anesthetic can be gradually reduced.^[9–11]^ No evidence suggests the guiding significance of burst-suppression in increasing and reducing anesthetic dose.^[9]^ In line with previous literature, we conclude that EEG burst-suppression and total suppression cannot provide guidance for the reduction of anesthetic (Figs. 3, 5, and 10).

The number of seizures rose apparently when the injection dose of propofol was increased (Figs. 6 and 8), while the number of seizures decreased apparently when propofol was gradually reduced (Figs. 7 and 9). Physiological waves and interictal epileptiform discharges recovered when propofol was stopped. By comparing the consequences of dose increase and decrease, we found that propofol aggravated the tonic seizures. As tonic seizures occur during natural sleep and after sleep induced by various narcotic drugs, the decrease of consciousness level induced by excessive sedation of narcotic drugs has been suggested as the reason for poor seizure control.^[12]^

Some benzodiazepines have been reported to induce tonic seizures.^[13]^ During the second sustained-state treatment, propofol was decreased when the dosage of clonazepam was increased. After the dosage of propofol was decreased, the number of seizures rose obviously in EEG signals, and GTCS and NCSE appeared. After the dosage of clonazepam was increased, the number of seizures was reduced in EEG signals, and the patient condition gradually improved. Therefore, benzodiazepines can be considered as the first-line drugs for the treatment of tonic status epilepticus.

The patient had a long course of illness, including mental retardation, spasticity at a young age and tonic seizure. Based on those symptoms, the diagnosis of LGS was established. Oxcarbazepine has been reported to aggravate LGS.^[14]^ However, during 5 years of taking oxcarbazepine, no deterioration of the patient condition was observed. In 2017, the International Anti-Epilepsy Alliance proposed the classification of seizures,^[15]^ pointing out that tonic seizures include both comprehensive and focal seizures. Considering the possibility of focal seizures in patients with previous tonic seizures, oxcarbazepine should not be considered as a contraindication for LGS.

The EEG signals showed full conduction low-amplitude fast-wave activity at the time of admission, with frequent seizures occurred at 34 hours after admission. The full conduction low amplitude fast wave activity could be a precursor of tonic status epilepticus.

Our case report has several potential limitations. The main cause of SE is not clear, and the withdrawal of antiepileptic drugs before admission may be the main cause of SE. Unfortunately, the patient failed to complete a brain MRI and lumbar puncture during hospitalization to rule out SE caused by immune diseases, which will be investigated in future research. We share the process of this case of high-dose anesthetics in the treatment of LGS, in order to provide clinical ideas and treatment experience for the diagnosis and treatment of similar symptoms. However, further case reports and research are needed to clarify the diagnosis and treatment of LGS.

## Author contributions

**Conceptualization:** Xiaoqian Yang, Guangjun Xu, Jingwei Du.

**Data curation:** Xiaoqian Yang, Yangyang Liang.

**Formal analysis:** Xiaoqian Yang, Guangjun Xu, Zonglei Chong.

**Investigation:** Xiaoqian Yang, Zonglei Chong, Lin Zhao.

**Methodology:** Xiaoqian Yang, Wei Chen.

**Project administration:** Xiaoqian Yang, Guangjun Xu, Wei Chen.

**Resources:** Xiaoqian Yang, Guangjun Xu, Wei Chen.

**Software:** Xiaoqian Yang, Zonglei Chong.

**Supervision:** Xiaoqian Yang, Guangjun Xu, Wei Chen.

**Validation:** Xiaoqian Yang, Wei Chen.

**Visualization:** Xiaoqian Yang.

**Writing – original draft:** Xiaoqian Yang, Zonglei Chong.

**Writing – review & editing:** Xiaoqian Yang, Zonglei Chong.
